# Defects in the Acquisition of Tumor-Killing Capability of CD8^+^ Cytotoxic T Cells in Streptozotocin-Induced Diabetic Mice

**DOI:** 10.1371/journal.pone.0109961

**Published:** 2014-11-12

**Authors:** Shu-Ching Chen, Yu-Chia Su, Ya-Ting Lu, Patrick Chow-In Ko, Pei-Yu Chang, Hung-Ju Lin, Hong-Nerng Ho, Yo-Ping Lai

**Affiliations:** 1 Department of Medical Research, National Taiwan University Hospital, Taipei, Taiwan; 2 National Laboratory Animal Center, National Applied Research Laboratories, Taipei, Taiwan; 3 Department of Internal Medicine, National Taiwan University Hospital, Taipei, Taiwan; 4 Department of Emergency Medicine, National Taiwan University Hospital, Taipei, Taiwan; 5 Department of Obstetrics and Gynecology, National Taiwan University, College of Medicine, Taipei, Taiwan; MRC National Institute for Medical Research, United Kingdom

## Abstract

Emerging evidences have shown that diabetes mellitus not only raises risk but also heightens mortality rate of cancer. It is not clear, however, whether antitumor CD8^+^ cytotoxic T lymphocyte (CTL) response is down-modulated in diabetic hosts. We investigated the impact of hyperglycemia on CTLs' acquisition of tumor-killing capability by utilizing streptozotocin-induced diabetic (STZ-diabetic) mice. Murine diabetes was induced by intraperitoneal injection of STZ (200 mg/kg) in C57BL/6 mice, 2C-T cell receptor (TCR) transgenic and P14-TCR transgenic mice. The study found that, despite harboring intact proliferative capacity measured with CFSE labeling and MTT assay, STZ-diabetic CD8^+^ CTLs displayed impaired effector functions. After stimulation, STZ-diabetic CD8^+^ CTLs produced less perforin and TNFα assessed by intracellular staining, as well as expressed less CD103 protein. Furthermore, adoptive transfer of STZ-diabetic P14 CD8^+^ effector cells showed an insufficient recruitment to the B16.gp33 melanoma and inadequate production of perforin, granzyme B and TNFα determined by immunohistochemistry in the tumor milieu. As a result, STZ-diabetic CD8^+^ effector cells were neither able to eliminate tumor nor to improve survival of tumor-bearing mice. Taken together, our data suggest that CD8^+^ CTLs are crippled to infiltrate into tumors and thus fail to acquire tumor-killing capability in STZ-diabetic hosts.

## Introduction

Diabetes and cancer are severe health concerns of worldwide significance. According to the estimation of World Health Organization, 347 million people worldwide have diabetes. In addition to severe complications caused by chronic hyperglycemia, epidemiological studies show that diabetic patients have higher risk of cancer [1]–[6], suggesting that diabetic patients carry impaired anti-tumor immunity.

CTL plays a cardinal role in anti-tumor defense. Upon activation, naïve CD8^+^ T cells are driven to clonal expansion and differentiation into the CTLs that exert cytokine production and tumor-lysis activity [7]–[10]. Glucose is essential fuel for T cell activation, proliferation, and acquisition of effector functions [11]–[15]. Chronic exposure to hyperglycemia may result in delayed response to antigen stimulation and failure to eliminate implanted ultraviolet-induced tumors [16]–[21]. The hypothesis is proposed that diabetes may cause defective CD8^+^ T cell responses that render diabetic hosts bearing poor tumor control. Nevertheless, two important questions remain unanswered. First, whether the diabetic condition hinders CD8^+^ T cell activation and differentiation into functional effector cells remains undefined. Second, it remains elusive in what extent of CD8^+^ T cells that are hampered by acute hyperglycemia.

STZ is used to induce diabetes by damaging pancreatic β-cells, resulting in insulin deficiency and consequently hyperglycemia [22], [23]. To investigate whether diabetes causes CD8^+^ T cell impairment, we used STZ-diabetic murine model to examine CD8^+^ T cell activation and differentiation both *in vitro* and *in vivo*. Furthermore, to evaluate anti-tumor immunity of STZ-diabetic CD8^+^ T cells, the effector functions at early and late differentiation stages were checked *in vitro*. Finally, we used murine melanoma model to assess tumor-killing capability of STZ-diabetic CD8^+^ T cells by monitoring tumor size and mice survival.

## Materials and Methods

### Ethical statements

All animal procedures in this study were followed guideline of the Use of Laboratory Animals published by National Taiwan University (NTU) and approved by Institutional Animal Care and Use Committee (IACUC) of College of Medicine and College of Public Health of NTU (Permit Number: 20100131). Mice were housed on a 12 h light–dark cycle, with the dark cycle occurring from 8:00 P.M. to 8:00 A.M in a specific pathogen-free environment of the animal center at NTU hospital. The mice enrolled in the study were monitored at least three times per week. All surgery was performed under sodium pentobarbital (30–90 mg/kg, intraperitoneal injection) anesthesia, and all efforts were made to minimize suffering. The humane endpoint criteria were set following IACUC guidelines, including the body weight loss of no more than 20% of pre-procedural weight, tumor size reaching 20 mm in diameter. At the end of experiment, mice were sacrificed by euthanasia with carbon dioxide gas inhalation. The spleens and tumors were collected after sacrifice of the mice.

### Mice

Male C57BL/6, B10.A, CD45.1, 2C and P14 TCR-transgenic mice at age of 6–8 weeks were obtained from animal center at NTU Hospital. The mice used in this study included more than eight mice in each group and all experiments were repeated for at least three independent times.

### Antibodies and reagents

Anti-mouse CD3 (clone 145-2C11) and anti-CD28 (clone 37.51) antibodies were prepared in our laboratory. DMEM, penicillin and streptomycin from GIBCO Inc. (Grand Island, NY, USA); fetal bovine serum (FBS) from HyClone Inc. (Logan, UT, USA); anti-mouse antibodies including FITC anti-CD3, -IFNγ and -granzyme B, PE anti-CD4, -CD19 and -perforin, PE-Cy5 anti-CD8 and APC anti-CD45.2 antibodies from eBioscience (San Diego, CA, USA); PE anti-TNFα and -CD103 antibodies from BioLegend (San Diego, CA, USA); STZ, Mitomycin C, LPS, MTT, phorbol myristic acid (PMA), Ionomycin and Brefeldin A from Sigma (St. Louis, MO, USA); CFSE from Molecular Probes (Eugene, OR, USA); QL9 (QLSPFPFDL) and KM9 (KAVTNFATM) peptides from AnaSpec, Inc. (San Jose, CA, USA) were purchased.

### Diabetes development

Diabetes was induced by intraperitoneal injection of STZ (200 mg/kg) into male mice as described previously [22], [23]. Blood glucose and weight were measured before and after STZ administration. Blood glucose level above 400 mg/dL was defined as diabetes.

### Cell preparation and culture conditions

Naïve (CD62L^hi^CD44^lo^) CD8^+^ T cells were obtained from spleens of mice by positive isolation of CD8^+^ T cells [24], and further by sorting on FACSAria (BD Bioscience, San Jose, CA, USA) through service provided by Flow Cytometric Analyzing and Sorting Core Facility (First Core Laboratory, NTU, College of Medicine). Cells culture was set up in DMEM containing 10% FBS and 5×10^−5^ M 2-Mercaptoethanol. To activate CD8^+^ T cells, the naïve cells were stimulated by anti-CD3/CD28 antibodies for the indicated time. For activating 2C TCR-transgenic (2C) CD8^+^ T cells, the cells were stimulated by mitomycin C-treated [25] LPS-stimulated B10.A B blasts [26] and QL9 peptide. Cells were grown in 5% CO_2_ humidified air at 37°C. For i*n vivo* priming, naïve 2C CD8^+^ T cells mixed with QL9-pulsed B10.A B blast cells were injected into the spleens of CD45.1 mice.

### Cell proliferation assays

#### CFSE (carboxyfluorescein succinimidyl ester) labeling

CFSE (5 mM) was added to the cells (10×10^6^ cells/mL) according to the manufacturer's instructions.

#### MTT (3-[4,5-dimethylthiazol-2-yl]-2,5-diphenyltetrazolium bromide) assay

Cells were incubated with MTT (1 mg/mL) for 4 hours. The formazan was solubilized by dimethyl sulfoxide and colorimetric absorbance was quantified by measuring optical density (OD) at 570 nm by a spectrophotometer (Tecan Group Ltd., Mannedorf, Switzerland).

### Intracellular cytokine staining

After 6-hour culture with PMA (10 ng/mL)/Ionomycine (1 µg/mL) and 4-hour culture with Brefeldin A (10 µg/mL), the cells were fixed and permeabilized with cytofix-cytoperm kit (BD Biosciences) and stained with specific antibodies according to the manufacturer's instructions.

### B16.gp33 melanoma model with adoptive transfer of P14 CD8^+^ effector cells

B16.gp33 cells derived from B16 melanoma cells and genetically modified to express gene encoding amino acid 33–41 of glycoprotein from lymphocytic choriomeningitis virus (LCMV) were kindly provided by Dr. Hanspeter Pircher [27] and cultured in DMEM supplemented with 10% FBS and 200 µg/mL G418. Following subcutaneous inoculation of B16.gp33 cells (1×10^6^ cells/mouse), the tumor diameter and survival of mice were recorded. P14 CTLs specific for LCMV gp33 in the context of H-2D^b^ were generated by activating the P14 naïve CD8^+^ T cells with mitomycin C-treated LPS-activated syngeneic B cell blasts and KM9 peptide, followed by harvest and cultured in recombinant human IL-2 (100 IU/mL)-containing medium as previously described [28]. The P14 CTLs in 1 X PBS (1×10^7^ cells/0.15 mL/mouse) were injected intravenously into the mice that had B16.gp33 tumor inoculation for 8 days.

### Detection of TNFα granzyme B and perforin in tumor-infiltrating lymphocytes

At 16 hours after transfer of P14 CTLs, the tumors were processed for cryosections and subjected to immunohistochemical staining by 2 µg/mL of FITC anti-granzyme B, PE anti-TNFα, PE anti-perforin and APC anti-CD45.2 antibodies.

### Statistical analysis

Experiments were performed independently for at least three times. The percentage of CD103^+^ cells in CD8^+^ T cells between three groups was analyzed by unpaired Student's *t*-test. The difference of relative distribution of immune cells and increased fold of tumor size between two groups was analyzed by Student's *t*-test. The survival difference between two groups was analyzed by logrank test. The general linear model was fitted for the unbalanced data to assess the difference of CD 45.2^+^ cell infiltration between two groups. Statistical significance was set at a *p* value of less than 0.05.

## Results

### STZ-diabetic mice and relative distribution of CD8^+^ T cells in peripheral lymphoid tissues

C57BL/6 male mice at the age of 6–8 weeks were administered with STZ intraperitoneally. Twenty days after STZ injection, blood glucose level was significantly increased (>400 mg/dL vs. non-diabetic control: 142.9±16.5 mg/dL, *p*<0.05) and weight was significantly decreased (18.5±1.7 g vs. 25.4±1.7 g, *p*<0.05) in STZ-diabetic mice (n = 22) compared to non-diabetic control mice (n = 15). The splenocytes of STZ-diabetic and C57BL/6 control mice were immune-phenotyped and analyzed by flow cytometry to study the relative distribution of CD8^+^ T cells in peripheral lymphoid tissues in diabetic condition, showing no significant difference between STZ-diabetic and control mice. To investigate whether the numbers of naïve CD62L^hi^CD44^l^°CD8^+^ T cells were changed in STZ-diabetic mice, the expression of CD62L and CD44 proteins in CD3^+^CD8^+^ T cells were further inspected. It showed that naïve CD8^+^ T cells still remained a significant population in the spleen of STZ-diabetic mice (data not shown).

### Proliferation of STZ-diabetic CD8^+^ T cells after activation

**Figure 1:**
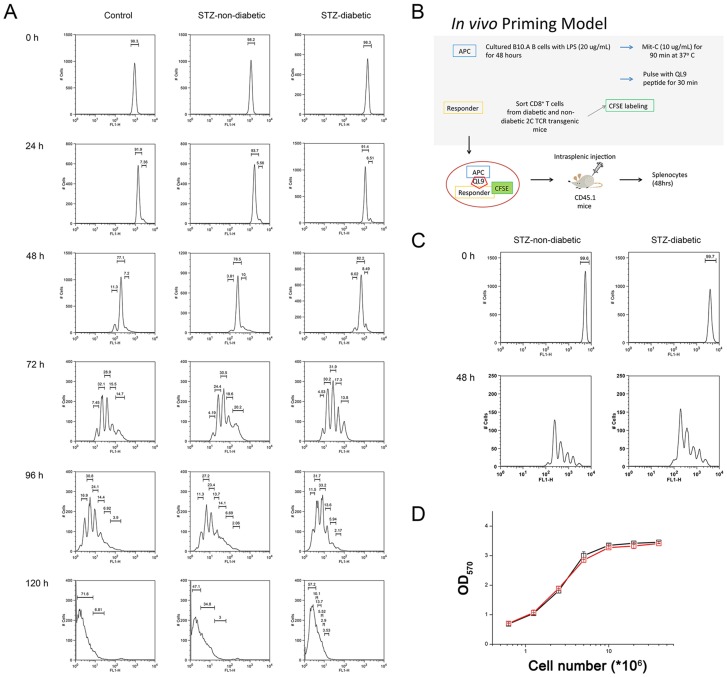
CD8^+^ T cell proliferation following stimulation. (A) CFSE-labeled STZ-diabetic, STZ-non-diabetic and control naïve CD8^+^ T cells from C57BL/6 mice were stimulated by anti-CD3/CD28 antibodies *in vitro* at the indicated time (24, 48, 72, 96 and 120 hours) and cell divisions were analyzed by flow cytometry. (B) *In vivo* CD8^+^ T cell priming model. (C) CFSE-labeled STZ-diabetic or STZ-non-diabetic 2C CD8^+^ T cells were co-administered with QL9-pulsed B10.A B blast cells into the spleen of healthy CD45.1 mice. Forty eight hours after priming, the splenocytes of CD45.1 mice were stained with PE anti-mouse CD45.2 antibody and cell divisions were analyzed by flow cytometry. (D) Five-day antigen-stimulated 2C CD8^+^ T cells from STZ-diabetic (red line) and STZ-non-diabetic (black line) mice were cultured in IL-2-containing medium for 24 hours, followed by MTT assay. Mit C, mitomycin C. APC, antigen-presenting cells. The data represent three independent experiments.

To investigate STZ-diabetic CD8^+^ T cell activation, CFSE-labeled naïve CD8^+^ T cells from STZ-diabetic and control C57BL/6 mice were stimulated by anti-CD3/CD28 antibodies *in vitro*, respectively, and harvested at indicated time for analyzing cell proliferation. It showed that, as control cells, STZ-diabetic CD8^+^ T cells had high proliferative capability (Figure 1A). To further study diabetic CD8^+^ T cell activation by specific antigenic peptide, the cell proliferation following *in vivo* priming was assessed (Figure 1B). It revealed that after priming for 48 hours, more than 50% of both STZ-diabetic and STZ-non-diabetic 2C CD8^+^ T cells had 3–4 cell divisions (Figure 1C), indicating that STZ-diabetic CD8^+^ T cells still attain proliferative capability upon stimulation *in vivo*. To further study the proliferative capability in late activated stage, 5-day stimulated 2C CD8^+^ T cells were cultured in IL-2-containing medium for 24 hours and cell proliferation was analyzed, revealing no significant difference between STZ-diabetic and STZ-non-diabetic groups (Figure 1D). Taken together, the results indicated that as non-diabetic cells, the STZ-diabetic CD8^+^ T cells possess proliferative potential.

### Effector function of STZ-diabetic CD8^+^ T cells

**Figure 2:**
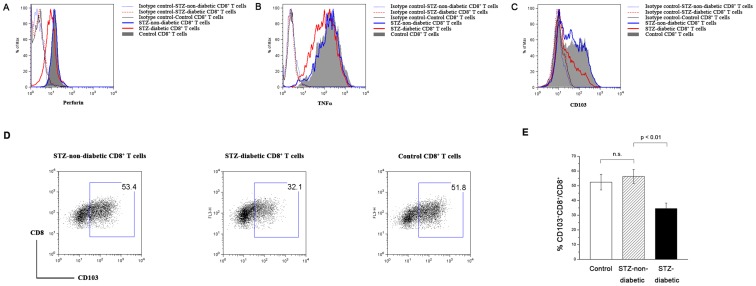
Effector functions of STZ-diabetic CD8^+^ T cells. (A, B) Naïve CD8^+^ T cells from STZ-diabetic (red line), STZ-non-diabetic (blue line) and control (gray-filled) C57BL/6 mice were stimulated by anti-CD3/CD28 antibodies *in vitro*. At 24 and 72 hours after stimulation, the production of perforin and TNFα was checked by intracellular staining and analyzed by flow cytometry. (C) At 120 hours of antigenic stimulation, 2C CD8^+^ T cells from STZ-diabetic (red line) and STZ-non-diabetic (gray-filled) mice were harvested and surface CD103 expression was checked and analyzed by flow cytometry. Staining of isotype control for the three experimental groups was shown: STZ-non-diabetic (blue dot), STZ-diabetic (red dash) and control (black line) mice. (D) CD103-positive population was gated in 5 day-stimulated 2C CD8^+^ T cells from STZ-non-diabetic, STZ-diabetic and control mice. (E) CD103-positive percentage in 5 day-stimulated 2C CD8^+^ T cells was analyzed from the three experimental groups. Data are representative of three independent experiments with three to five mice per time point.

The anti-CD3/CD28-stimulated CD8^+^ T cells from STZ-diabetic and non-diabetic control C57BL/6 mice were harvested at indicated time for checking the effector function. Significant production of IL-2, IFNγ and Granzyme B was observed in all groups (data not shown). However, STZ-diabetic CD8^+^ T cells produced less perforin and TNFα at 24–72 hours after stimulation (Figures 2A and 2B), indicating an impaired effector function at early differentiation stage. Besides, expression of CD103protein was significantly lower in STZ-diabetic 2C CD8^+^ T cells on day 5 following stimulation (Figure 2C). Of note, fewer CD103^+^ cells were present in STZ-diabetic CD8^+^ T cell population (Figures 2D and 2E), implying accumulation deficit of diabetic CTLs in tumor and thereby compromising anti-tumor immunity.

### Anti-tumor immunity of STZ-induced diabetic CD8^+^ T cells

**Figure 3:**
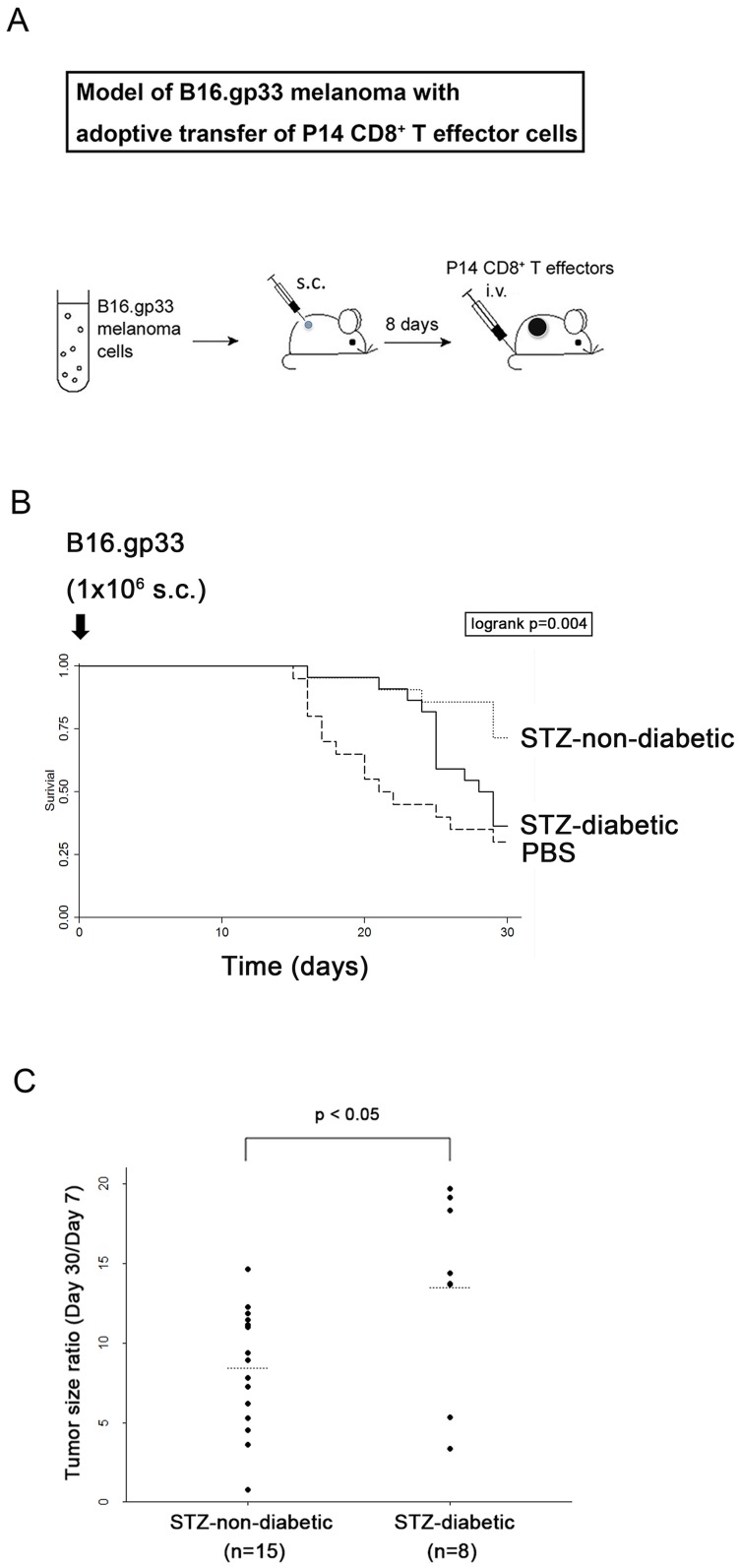
Impaired anti-tumor immunity of STZ-diabetic CTLs. (A) Murine model of B16.gp33 melanoma with adoptive transfer of P14 CD8^+^ T effector cells. (B) After adoptive transfer of STZ-diabetic (n = 22) or STZ-non-diabetic (n = 21) P14 CD8^+^ T effector cells into B16.gp33 melanoma-bearing C57BL/6 mice, the survival time of the mice was recorded. Tumor-bearing mice with PBS but not T cells injection were considered as the controls (n = 20). (C) Tumor size of the mice that survived on day 30 after tumor inoculation. The increased fold of tumor size was calculated as tumor size at day 30 divided by that at day 7.

**Figure 4:**
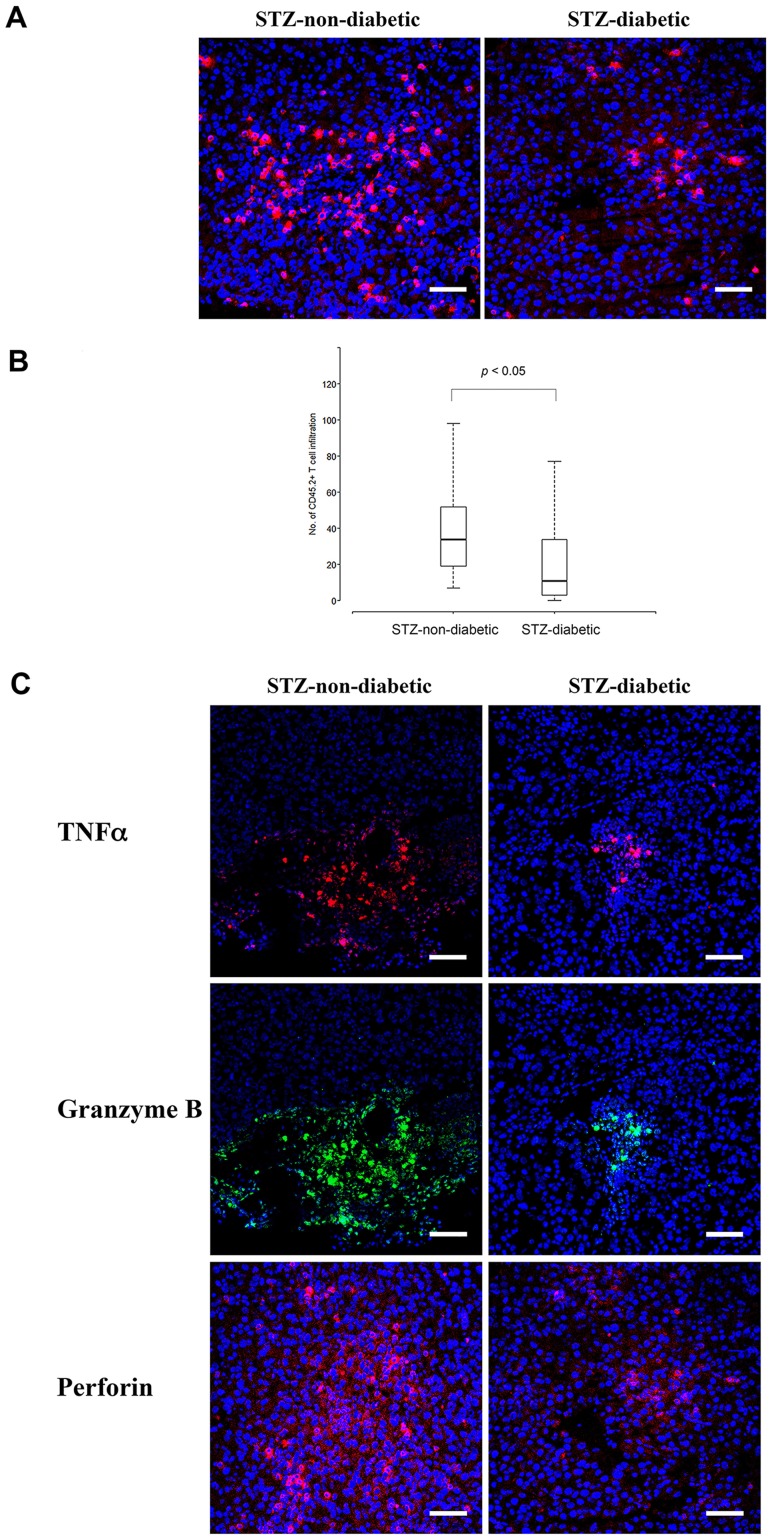
Infiltration and cytotoxicity of STZ-diabetic CTLs in tumor. (A, B) Assessment of the tumor-specific CTL infiltration in the tumor. Sixteen hours after adoptive transfer of STZ-diabetic or STZ-non-diabetic P14 CD8^+^ T effector cells into tumor-bearing CD45.1 mice, the tumors were processed for cryosections and stained by APC anti-mouse CD45.2 antibody and Hoechst 33342. (C) The production of TNFα, granzyme B and perforin by STZ-diabetic or STZ-non-diabetic P14 CTLs was monitored by immunostaining the tumors with specific antibodies and Hoechst 33342, and were visualized by a Zeiss LSM 510 confocal microscope. Scale bar: 50 µm.

To investigate tumor-killing capability of STZ-diabetic CD8^+^ T cells *in vivo*, B16-gp33 melanoma cells were subcutaneously inoculated, followed by adoptive transfer of the tumor-specific P14 CD8^+^ effector cells intravenously (Figure 3A). All the mice developed tumor and 70% of the PBS control group (14/20) died of tumor burden whereas, only 29% of the mice with adoptive transfer of STZ-non-diabetic P14 CD8^+^ effector cells died (6/21) during the experimental observation period. It showed that adoptive transfer of STZ-non-diabetic P14 CD8^+^ effector cells caused a prolonged survival from 36% (8/22 mice of STZ-diabetic group) to 71% (15/21 mice of STZ-non-diabetic group) of tumor-bearing mice on 30 days after tumor inoculation (Figure 3B, *p*<0.01). By contrast, transfer of the STZ-diabetic P14 CD8^+^ effector cells did not show beneficial effect on survival (8/22 mice of STZ-diabetic group vs. 6/20 mice of PBS injection group). Furthermore, smaller tumor size was revealed in STZ-non-diabetic P14 CD8^+^ effector cells-treated group (Figure 3C, *p*<0.05). Taken together, STZ-diabetic CD8^+^ T cells are defective in tumor eradication *in vivo*. To elucidate the tumor-infiltrating efficacy of tumor-specific T cells, the tumor was removed and intra-tumor CD45.2^+^ cells was analyzed by immunohistochemical staining at 16 hours after adoptive transfer of P14 CD8^+^ effector cells (CD45.2^+^) into tumor-bearing CD45.1 mice, (Figure 4A). Significantly fewer CD45.2^+^ cells were found in the tumor of mice receiving STZ-diabetic P14 CD8^+^ effector cells than STZ-non-diabetic group (Figure 4B), STZ-diabetic: 24.8, 95% confidence interval (CI), 14.8–34.8; STZ-non-diabetic: 43.7, 95% CI, 34.4–52.9; *p<0.01*. the difference: 18.9, 95% CI, 5.3–32.5; *p<0.01*). Moreover, there were significantly fewer perforin-, granzyme B- and TNFα-producing cells in the tumor of mice receiving STZ-diabetic P14 CD8^+^ effector cells than STZ-non-diabetic group (Figure 4C). Therefore, STZ-diabetic P14 T cells had impaired effector function and were ineffective against tumor burden *in vivo*.

## Discussion

Diabetic patients have an increased incidence of cancers [1]–[6], suggesting that diabetes hampers anti-tumor CTL function. Considerable evidence has accumulated supporting the importance of abundant tumor-infiltrating CTLs for better outcomes in various types of cancers [29]–[31]. Therefore, appropriate intra-tumor migration of CTLs is a prerequisite for antitumor surveillance. Our data showed that adoptive transfer of STZ-diabetic CD8^+^ effector cells resulted in fewer tumor-infiltrating T cells as well as less production of perforin, Granzyme B and TNFα *in situ*. It is plausible that, in addition to defective cytotoxicity, the intra-tumor migration of STZ-diabetic CTLs is impeded.

Recent reports have shown that TNFα can promote influx of tumor-reactive T cells by remodeling intra-tumor vessels [32]–[34], and thereby exerts a local immunomodulatory function in tumor microenvironment. Our study showed that the less production of intra-tumor TNFα in STZ-diabetic CD8^+^ T cells-treated mice may lead to insufficient infiltration of tumor-reactive CTLs. Furthermore, anti-tumor defense requires recognition of tumor antigens by CTLs' TCRs and strengthening CTL/tumor cell contacts by LFA-1-ICAM-1 and/or CD103-E-cadherin interaction [35]–[39]. This firm adhesion constructed by CD103-E-cadherin interaction is crucial for CTLs to kill tumor cells especially when tumors do not express ICAM-1 [37], [39]. It ensures tumor killing by promoting the maturation of immunological synapse and polarized release of cytokine and lytic granules. Thus, significantly less expression of CD103 protein on STZ-diabetic CTLs revealed in our study may cripple CTLs' intratumor migration and firm retention in tumors, leading to insufficient cytokine production and cytotoxicity toward tumor cells.

The findings from the current study strongly suggest that the effect of diabetes on CD8^+^ T cell function should be reconsidered more precisely. Overall, these results provide values for identifying STZ-induced diabetes may hamper the CTL function as a result of impaired cell differentiation. The enfeebled CTLs with inadequate CD103 expression show ineffective tumor infiltration and insufficient production of the cytotoxic mediators. The elucidation of how effector functions of STZ-diabetic CD8^+^ T cells are impeded will optimize strategies for advancing tumor-killing capability and inducing protective antitumor immunity in diabetic hosts.

## Supporting Information

Checklist S1 **The Arrive Guidelines Checklist for Animal Research reports** ***in vivo*** **experiments of this study.**
(PDF)
